# Construction of nomogram model of poor prognosis for patients newly diagnosed with brain metastasis from non-small cell lung cancer based on clinical pathology and prognostic scores

**DOI:** 10.3389/fonc.2025.1487126

**Published:** 2025-03-07

**Authors:** Zengliang Li, Xiaoyue Wang, Guodong Ma

**Affiliations:** ^1^ Department of Thoracic Surgery, Nanjing Chest Hospital, Nanjing, China; ^2^ Department of Thoracic Surgery, Affiliated Nanjing Brain Hospital, Nanjing Medical University, Nanjing, China; ^3^ Department of Respiratory Medicine, Nanjing Chest Hospital, Nanjing, China; ^4^ Department of Respiratory Medicine, Affiliated Nanjing Brain Hospital, Nanjing Medical University, Nanjing, China

**Keywords:** clinical pathology, prognostic score, non-small cell lung cancer, brain metastases, unfavourable prognosis

## Abstract

**Objective:**

To explore non-small cell lung cancer (NSCLC) patients with new diagnosis of brain metastasis and construct Logistic regression model based on clinical pathology and prognosis score, and verify.

**Methods:**

A total of 158 patients newly diagnosed with brain metastasis in NSCLC were retrospectively selected from March 2020 to April 2022. The clinical data of patients were collected, and Logistic regression analysis was used to analyze the influencing factors of poor prognosis for newly diagnosed NSCLC with brain metastasis.

**Results:**

The results of univariate analysis showed that the clinical pathological features including NLR>2.94, abnormal CEA, mediastinal lymph node metastasis, symptomatic treatment with therapeutic method, extracranial metastasis and GPS1-2 score were associated with the survival and prognosis of patients with newly diagnosed brain metastasis from NSCLC (*P* < 0.05). Multivariate Logistic regression analysis showed that NLR>2.94, mediastinal lymph node metastasis, CEA abnormality, extracranial metastasis, and newly diagnosed NSCLC with GPS1-2 score were independent risk factors for poor prognosis of brain metastasis (*P* < 0.05). Internal verification using the Bootstrap method showed that the predicted curve fitted well with the standard model curve, with the average absolute error of 0.029. The ROC curve result showed that the AUC was 0.887, and the 95%CI was 0.782–0.905, with the corresponding specificity and sensitivity of 90.50% and 80.00%, respectively. This indicates that the prediction accuracy of this Nomogram model is good.

**Conclusion:**

NLR, mediastinal lymph node metastasis, CEA, extracranial metastasis and GPS are risk factors for poor prognosis of newly diagnosed brain metastasis in NSCLC. The risk factor model constructed based on these risk factors has excellent prediction value for the poor prognosis of newly diagnosed brain metastasis in NSCLC. In order to reduce the risk of newly diagnosed brain metastasis in NSCLC and improve the prognosis, targeted preventive measures are taken against the above risk factors in clinical practice.

## 1 Introduction

Lung cancer, particularly non-small cell lung cancer (NSCLC), is the leading cause of cancer-related deaths, accounting for 11.4% of global cancer cases, with NSCLC representing approximately 85% of lung cancer diagnoses (1). Brain metastasis is one of the common distant metastasis sites of NSCLC, and the incidence rate is about 20% to 40%. Brain metastasis will not only lead to nervous system symptoms such as headache, vomiting, and limb weakness in patients, but also seriously affect the quality of life and prognosis of patients (2). In recent years, it has been found (3, 4) that the mechanism of NSCLC brain metastasis involves multiple molecular and cellular factors, and EGFR mutations play an important role in NSCLC brain metastasis. EGFR mutations promote the invasion and brain metastasis of NSCLC cells by activating downstream signaling pathways such as the MET gene and STAT3 signaling pathway; Immune cells such as astrocytes and macrophages in the tumor microenvironment help tumor cells invade and colonize the brain by secreting inflammatory factors (such as IL-8, IL-6, etc.) and matrix metalloproteinases (such as MMP2, MMP9). Patients newly diagnosed with brain metastasis by NSCLC will suffer from increased intracranial pressure and cross-line paralysis, which will seriously damage the quality of life of patients and shorten their survival expectations. Although the survival time of patients newly diagnosed with brain metastasis by NSCLC is extended to a certain extent by the intervention strategy of surgery combined with multiple treatments, the overall prognosis is still poor (5, 6). Therefore, studying the influencing factors of poor prognosis in patients with brain metastasis will help to provide the basis for clinical treatment decisions, improve the prognosis of patients and improve the quality of life. Scholars in China and abroad have conducted a large number of studies on the factors influencing the poor prognosis of patients with NSCLC brain metastases and found that factors such as age, gender, pathological type, molecular biological characteristics, the number of brain metastases, and treatment method are closely related to the prognosis of patients (7, 8). However, there is still a lack of a unified prognosis evaluation model to facilitate clinicians to conduct individualized treatment and prognosis evaluation for patients with brain metastasis. Therefore, constructing a simple and effective prognosis prediction model has important clinical application value. In recent years, with the advancement of medical technology and the deepening of research, more and more studies have begun to focus on predictive models for NSCLC brain metastasis. Especially in the exploration of prognostic markers, scholars have made significant progress. For example, studies have found that NLR (neutrophil to lymphocyte ratio) is not only an indicator of inflammatory status, but also plays an important role in predicting the prognosis of various malignant tumors (7). In addition, GPS (Glasgow Prognostic Score) has also been proven to be an important predictor of prognosis for various malignant tumor patients (8). The purpose of this study was to explore the influencing factors of poor prognosis in patients with newly diagnosed brain metastases from NSCLC and to construct an nomogram model in order to provide the basis for clinical decision-making.

## 2 Data and methods

### 2.1 General information

A total of 158 patients with newly diagnosed brain metastases from NSCLC who were treated in our hospital from March 2020 to April 2022 were retrospectively selected and followed up for two years. This study was approved by the Hospital Ethics Committee.

### 2.2 Inclusion and exclusion criteria

Inclusion criteria: Patients who met the diagnostic criteria of NSCLC (9), were diagnosed with NSCLC by histology or cytology, and showed ≥3 newly diagnosed brain metastatic lesions after treatment. All patients had no autoimmune disease and no non-tumor-related infections. Exclusion criteria: Patients with cerebrovascular diseases, previous secondary malignant tumors, or those who were lost to follow-up or had incomplete clinical data. Patients with significant comorbidities (e.g., severe cardiovascular disease, chronic kidney disease) that could independently affect survival were also excluded to ensure the representativeness of the sample.

### 2.3 Methodology

Data collection: general data of that patient include age, smoking history, pathological type, karnofsky’s score (KPS), stage (TNM), neutrophil to lymphocyte ratio (NLR), lactate dehydrogenase (LDH), D- dimer (D-D), carcinoembryonic antigen (CEA), carbohydrate antigen (CA125), gene mutation, cytokeratin 19 fragment antigen (Cyfra-211) obtain through an electronic medical record system; Proliferation markers (Ki-67), mediastinal lymph node metastasis, neurological symptoms, brain metastasis pattern, number of brain metastases, extracranial metastasis, treatment, Glasgow outcome score (GPS), and follow-up analysis of two years.

LDH: reference range 120-250 U/L; D-D: reference range 0-1 ug/mL; CEA: reference range 0-5 ng/mL; CA125 reference range 0-36 U/mL; Cyfra-211: Reference range 0-2.08ng/ml. The reference ranges of the above indicators were within the normal range, and the increase or decrease was abnormal.

In the evaluation of the biological characteristics of the patient, the expression levels of NLR, LDH, D-D, Ki-67, CEA, CA125 were determined by immunohistochemistry. All testing procedures are strictly in accordance with the operation instructions provided by the corresponding kit.

TNM staging: The latest American Joint Committee on Cancer (AJCC) version 8 staging system was used.

Brain metastasis: The starting point of the study was the moment when the patient was first diagnosed with NSCLC, and the subsequent time points when the diagnosis of brain metastasis was confirmed as the end point. The interval of brain metastasis was calculated. Brain metastases that occur more than three months after the diagnosis of NSCLC are defined as metachronous brain metastases; Conversely, brain metastases that are detected within 3 months of a definite diagnosis of NSCLC are considered to be simultaneous brain metastases.

### 2.4 Establishment and validation of diagnostic model

The nomogram prediction model was constructed using the R statistical software platform, and the C index was calculated using the Bootstrap resampling technique. The prediction model was constructed by plotting the receiver operating characteristic curve (ROC) and based on the survival data. In the feature screening stage, the most predictive features were selected using Logistic regression analysis for regression analysis, aiming to reveal the potential factors leading to poor prognosis and integrate these key factors into our model. The visual form shows the structure and prediction ability of the prediction model. In order to fully verify the model performance, assessment methods such as area under curve (AUC) and calibration curve were used to constitute a comprehensive evaluation system for the accuracy, reliability and clinical applicability of the nomogram model.

Variables for inclusion in the logistic regression model were selected based on their clinical relevance and statistical significance in univariate analysis. Variables with a p-value < 0.05 in univariate analysis were included in the multivariate model. Multicollinearity among the variables was assessed using the variance inflation factor (VIF), with a threshold of VIF < 5 indicating no significant multicollinearity. All selected variables met this criterion, ensuring the robustness of the model.

### 2.5 Statistical analysis

Enumeration data were expressed as [cases (%)], and χ2 test was used. The measurement data were tested for normal distribution and conformed to normal distribution, all in the form of ( x ± s ). The measurement data between two groups were tested by t test. SPSS 23.0 software was used for statistical data analysis. *P*<0.05 indicated that the difference was statistically significant.

## 3 Results

### 3.1 Kaplan Meier survival curve analysis

**Figure 1:**
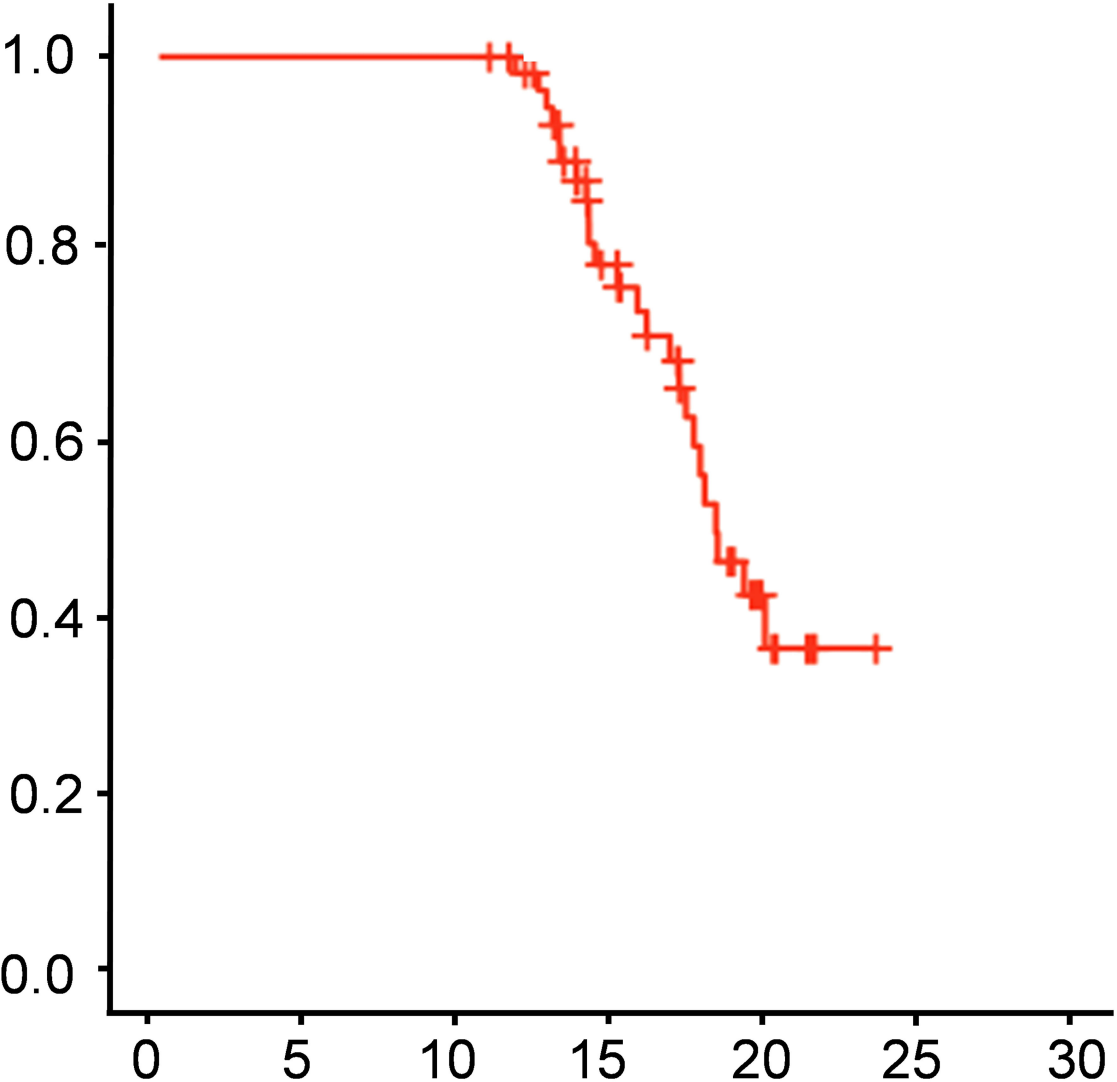
Kaplan-Meier curve.

**Figure 2:**
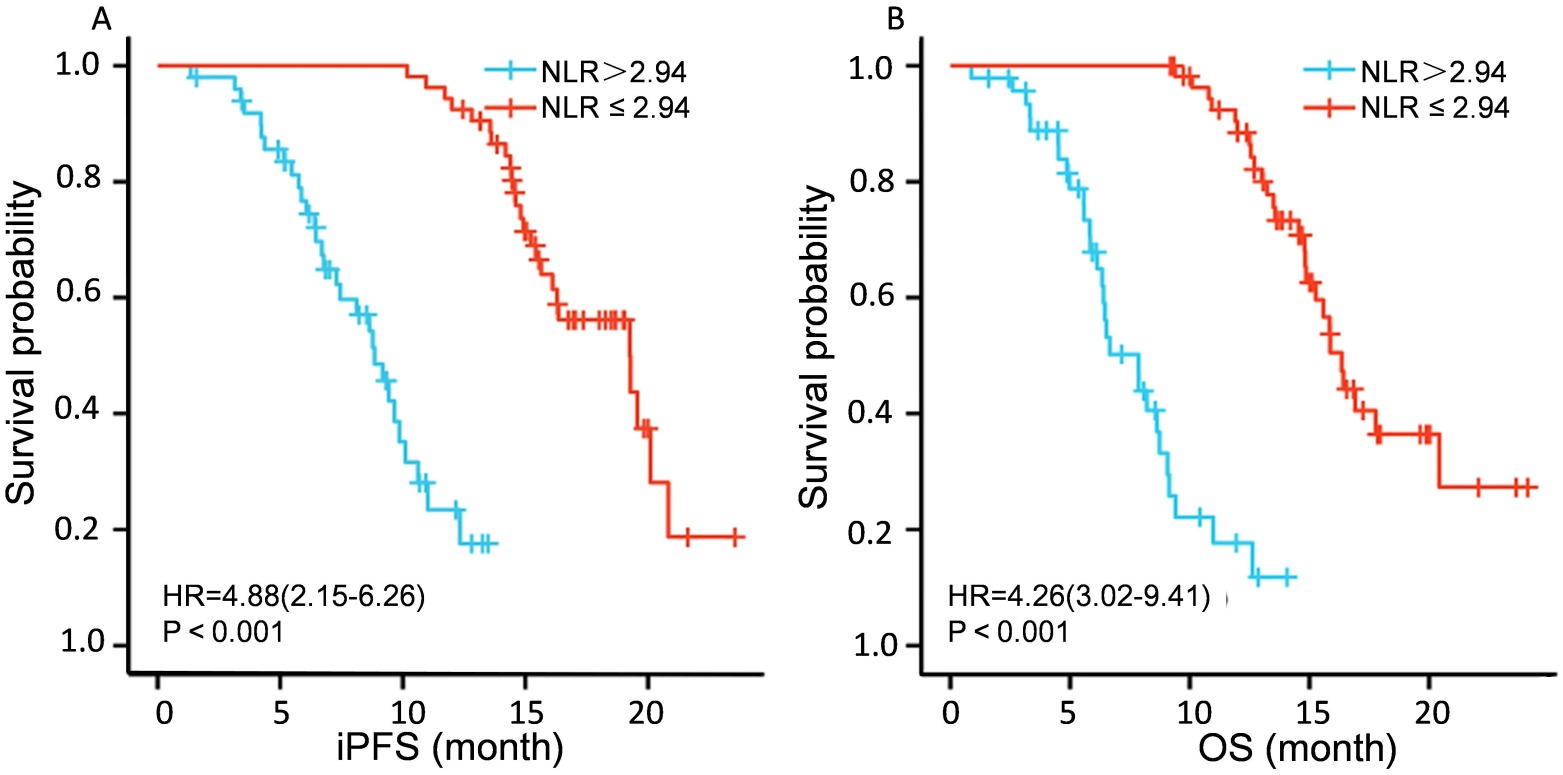
PFS and OS analysis of patients with different NLR expressions. **(A)** shows a comparison of iPFS among patients with different NLR expression levels. **(B)**shows a comparison of OS expression among different NLRs.

The two-year intracranial progression-free survival (iPFS) rate was 58.2%, and the median overall survival (OS) was 15 months. **Figure 1** shows the Kaplan-Meier curve for overall survival, illustrating the survival outcomes of the cohort over the two-year follow-up period. Draw Kaplan Meier survival curves to compare the intracranial progression free survival (iPFS) and overall survival (OS) of patients with NLR>2.94 and NLR ≤ 2.94, The curves demonstrate significant divergence, with the NLR > 2.94 subgroup exhibiting markedly shorter survival (P < 0.001 for both iPFS and OS), See **Figure 2**.

### 3.2 Single factor analysis of clinical data of two groups

Univariate analysis showed that the clinical pathological features including NLR>2.94, abnormal CEA, mediastinal lymph node metastasis, symptomatic treatment with therapeutic method, extracranial metastasis and GPS1-2 score were associated with the survival and prognosis of patients with newly diagnosed brain metastasis from NSCLC (*P* < 0.05). See **Table 1**

**Table 1:** Analysis of clinical data of patients in two groups [Cases (%)].

Clinical pathological features	Number of cases	Median survival (months)	Survival (%)			*χ2*	*P*
			6 months	1 year	2 years		
gender							
man	70	20	82.86 (58/70)	65.71 (46/70)	17.14 (12/70)	0.489	0.484
woman	88	14	72.72 (64/88)	51.13 (45/88)	21.59 (19/88)		
Age (years)							
≤60	37	19	83.78 (31/37)	64.86 (24/37)	21.62 (8/37)	0.056	0.813
>60	121	15	75.20 (91/121)	55.37 (67/121)	19.83 (24/121)		
Smoking history							
without	76	14	72.36 (55/76)	52.63 (40/76)	21.05 (16/76)	0.058	0.809
have	82	18	81.70 (67/82)	60.97 (50/82)	19.51 (16/82)		
KPS (points)							
≤90	109	14	75.22 (82/109)	50.45 (55/109)	20.18 (22/109)		
>90	49	19	81.63 (40/49)	71.42 (35/49)	20.40 (10/49)		
Pathological type							
squamous carcinoma	7	8	85.71 (6/7)	0.00 (0/7)	0.00 (0/7)	3.502	0.320
glandular cancer	143	15	76.92 (110/143)	58.04 (83/143)	20.97 (30/143)		
adenosquamous carcinoma	4	8	100.00 (4/4)	50.00 (2/4)	50.00 (2/4)		
Large cell carcinoma	4	21	100.00 (4/4)	100.00 (4/4)	0.00 (0/4)		
T staging							
T1-3	94	15	76.59 (72/94)	53.19 (50/94)	17.02 (16/94)	2.098	0.148
T4	64	17	79.68 (51/64)	62.50 (40/64)	26.56 (17/64)		
N staging							
NO-1	58	18	79.31 (46/58)	53.44 (31/58)	18.96 (11/58)	0.094	0.759
N2-3	100	15	76.00 (76/100)	59.00 (59/100)	21.00 (21/100)		
NLR							
≤2.94	76	18	88.15 (67/76)	64.47 (49/76)	28.94 (22/76)	6.854	0.008
>2.94	82	13	67.07 (55/82)	50.00 (41/82)	12.19 (10/82)		
LDH (IU/L)							
normal	112	17	81.25 (91/112)	60.71 (68/112)	20.53 (23/112)	0.019	0.890
abnormal	46	12	67.39 (31/46)	47.82 (22/46)	19.56 (9/46)		
D-D (mg/L)							
normal	86	19	88.37 (73/86)	65.11 (56/86)	25.58 (22/86)	2.518	0.113
abnormal	72	12	69.44 (50/72)	47.22 (34/72)	15.27 (11/72)		
CEA (μg/L)							
normal	43	18	76.74 (33/43)	62.79 (27/43)	9.30 (4/43)	5.221	0.022
abnormal	115	16	78.26 (90/115)	54.78 (63/115)	26.08 (30/115)		
CA125 (U/ml)							
normal	80	20	85.00 (68/80)	62.50 (50/80)	17.50 (14/80)	0.462	0.497
abnormal	78	14	69.23 (54/78)	51.28 (40/78)	21.79 (17/78)		
Cyfra-211 (U/ml)							
normal	27	23	81.48 (22/27)	66.66 (18/27)	25.92 (7/27)	0.501	0.479
abnormal	131	15	76.33 (100/131)	54.96 (72/131)	19.84 (26/131)		
gene mutation							
positive	33	21	81.81 (27/33)	63.63 (21/33)	21.21 (7/33)	0.024	0.878
negative	125	16	76.00 (95/125)	56.00 (70/125)	20.00 (25/125)		
KI-67 (%)							
≤30%	55	15	70.90 (39/55)	54.54 (30/55)	20.00 (11/55)	0.003	0.953
>30%	103	18	81.55 (84/103)	58.25 (60/103)	20.38 (21/103)		
Mediastinal lymph node metastasis							
without	85	14	81.17 (69/85)	52.94 (45/85)	12.94 (11/85)	7.247	0.007
have	73	17	73.97 (54/73)	63.01 (46/73)	30.13 (22/73)		
Neurological symptoms							
without	66	19	86.36 (57/66)	65.15 (43/66)	24.24 (16/66)	0.773	0.379
have	92	13	70.65 (65/92)	50.00 (46/92)	18.47 (17/92)		
Brain metastasis pattern							
heterochrony	57	14	75.43 (43/57)	50.87 (29/57)	19.29 (11/57)	0.136	0.712
synchronism	101	17	78.21 (79/101)	59.40 (60/101)	21.78 (22/101)		
Number of brain metastases (a)							
≤3	61	20	83.60 (51/61)	62.29 (38/61)	26.23 (16/61)	1.717	0.190
>3	97	23	74.22 (72/97)	53.60 (52/97)	17.52 (17/97)		
Extracranial metastasis							
without	64	23	85.93 (55/64)	70.31 (45/64)	31.25 (20/64)	8.055	0.005
have	94	12	71.27 (67/94)	47.87 (45/94)	12.76 (12/94)		
Treatment							
Symptomatic treatment	16	4	25.00 (4/16)	6.25 (1/16)	0.00 (0/16)	11.950	0.003
Simple treatment	43	12	64.12 (28/43)	44.18 (19/43)	9.23 (4/43)		
Combination therapy	99	20	90.90 (90/99)	70.70 (70/99)	29.29 (29/99)		
GPS (minutes)							
0	65	15	87.50 (56/64)	68.75 (44/64)	33.85 (22/65)	11.225	0.001
1-2	93	16	73.11 (68/93)	49.46 (46/93)	11.82 (11/93)		

### 3.3 Single factor analysis of influencing factors of PABC

Whether brain metastasis newly diagnosed as NSCLC has poor prognosis is taken as the dependent variable, and the statistically different indicators in **Table 1** are taken as the independent variable. The assignment results are shown in **Table 2**. Multivariate Logistic regression analysis showed that NLR>2.94, mediastinal lymph node metastasis, CEA abnormality, extracranial metastasis, and newly diagnosed NSCLC with GPS1-2 score were independent risk factors for poor prognosis of brain metastasis (*P* < 0.05). See **Table 3**

**Table 2:** valuation of independent variables.

Variable	Assignment condition	
X1	NLR	1=>2.94, 0=≤2.94
X2	CEA	1= abnormal, 0= normal
X3	Mediastinal lymph node metastasis	1= yes, 0= none
X4	Extracranial metastasis	1= yes, 0= none
X4	Treatment	1= symptomatic treatment, simple treatment, 0= combined treatment
X5	GPS	1 = 1-2 points, 0 = 0 points
X6	Prognosis	1= poor prognosis, 0= good prognosis

**Table 3:** Factors influencing poor prognosis of newly diagnosed brain metastases in NSCLC by multivariate Logistic regression analysis.

index	*B value*	*SE value*	*Wald value*	*P value*	*OR value*	95%CI
NLR	0.866	0.342	6.442	0.012	2.375	1.217~4.631
Treatment	0.852	0.214	14.702	0.071	2.261	1.215~3.025
Extracranial metastasis	0.989	0.374	6.991	0.001	2.688	1.293~5.598
Mediastinal lymph node metastasis	1.884	0.338	7.197	0.001	6.574	3.396~12.731
CEA	0.614	0.234	6.903	0.008	1.848	1.168~2.925
GPS	0.521	0.249	4.082	0.007	1.672	1.031~5.914

### 3.4 Establishment of nomogram prediction model

**Figure 3:**
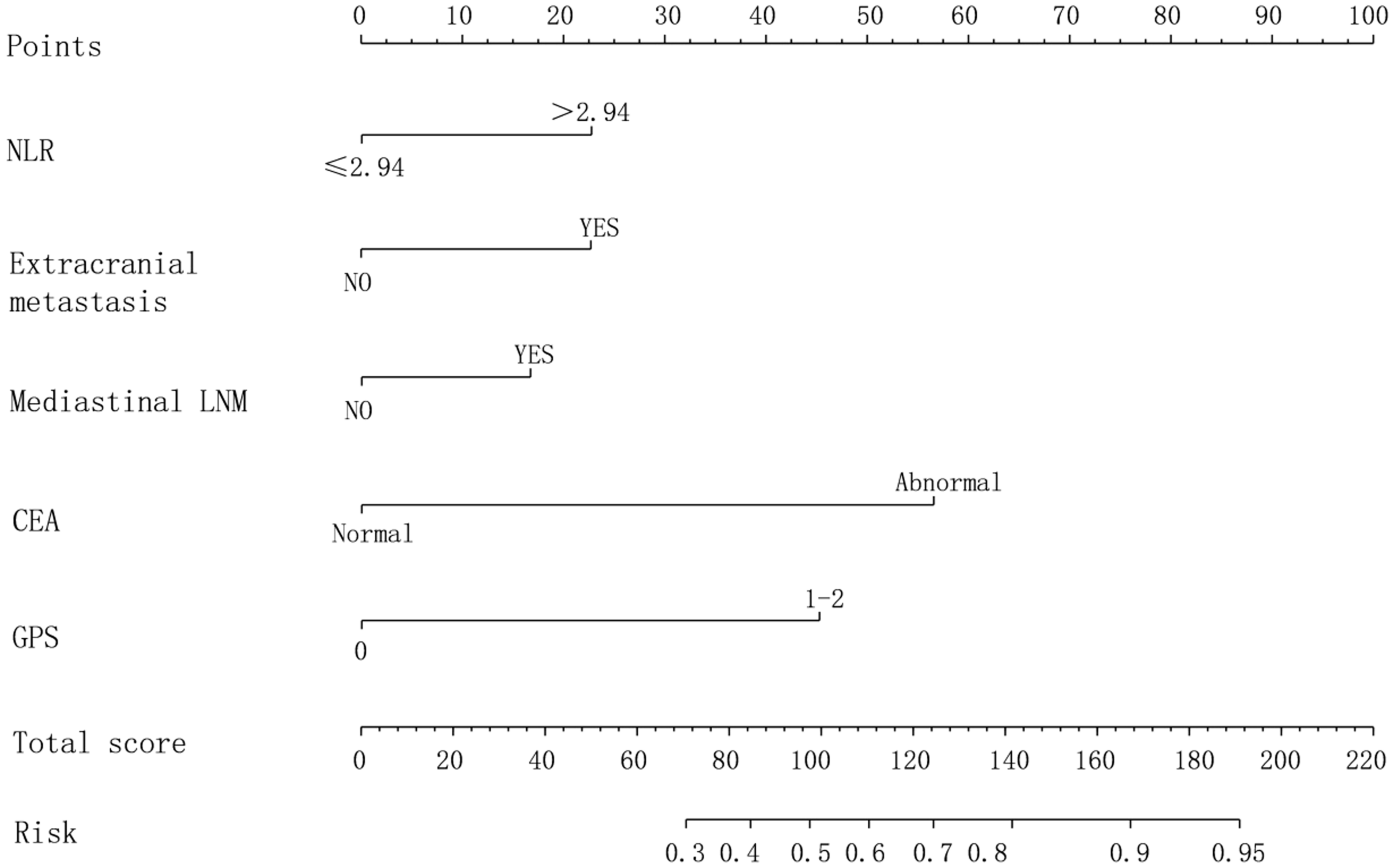
Nomogram prediction model for newly diagnosed brain metastasis in NSCLC with poor prognosis.

Based on the results of Logistic regression analysis, a risk prediction model for newly diagnosed brain metastasis in NSCLC was constructed, as shown in **Figure 3**.

### 3.5 Validation of nomogram prediction model

**Figure 4:**
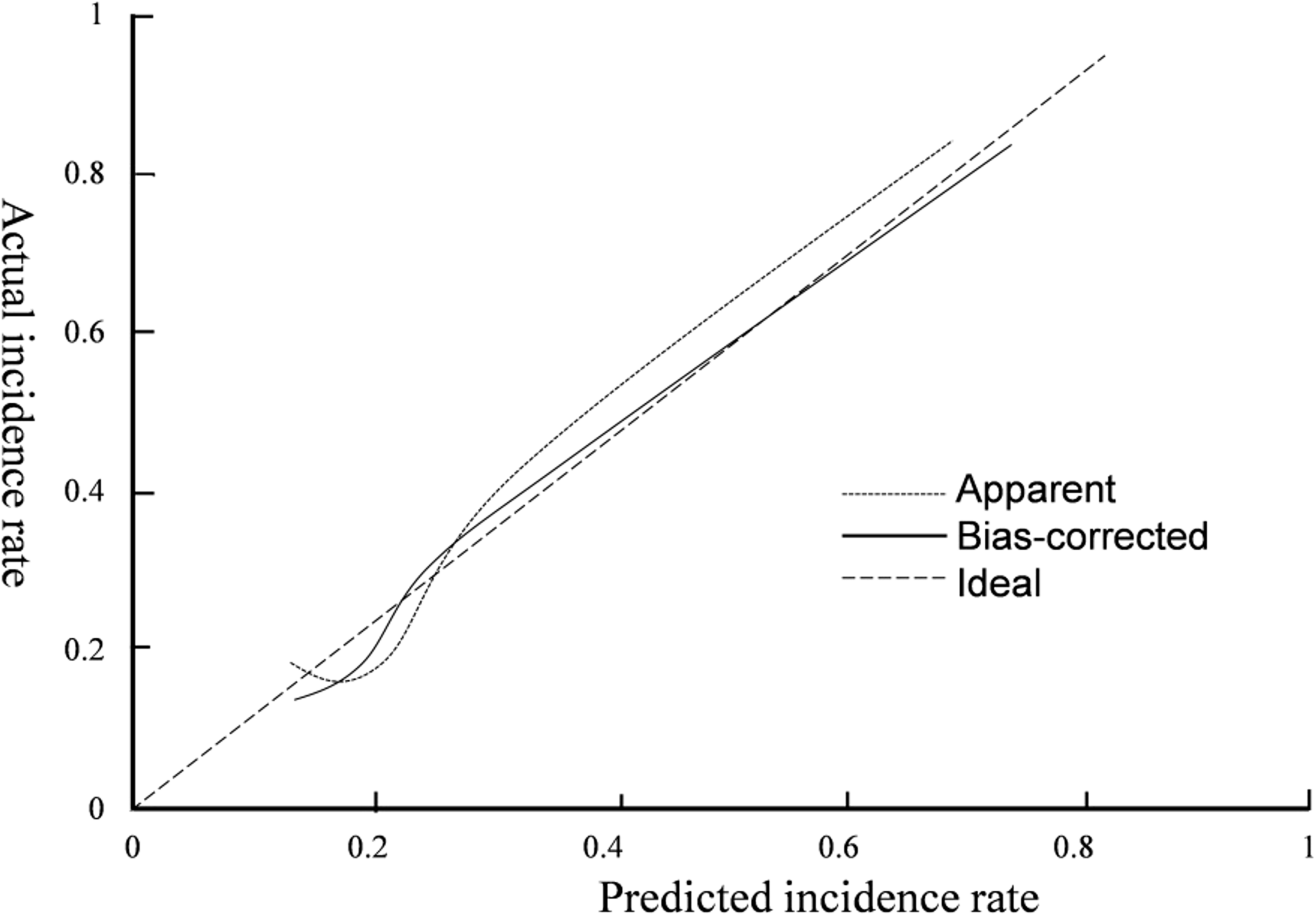
Calibration curve of Nomogram prediction model.

**Figure 5:**
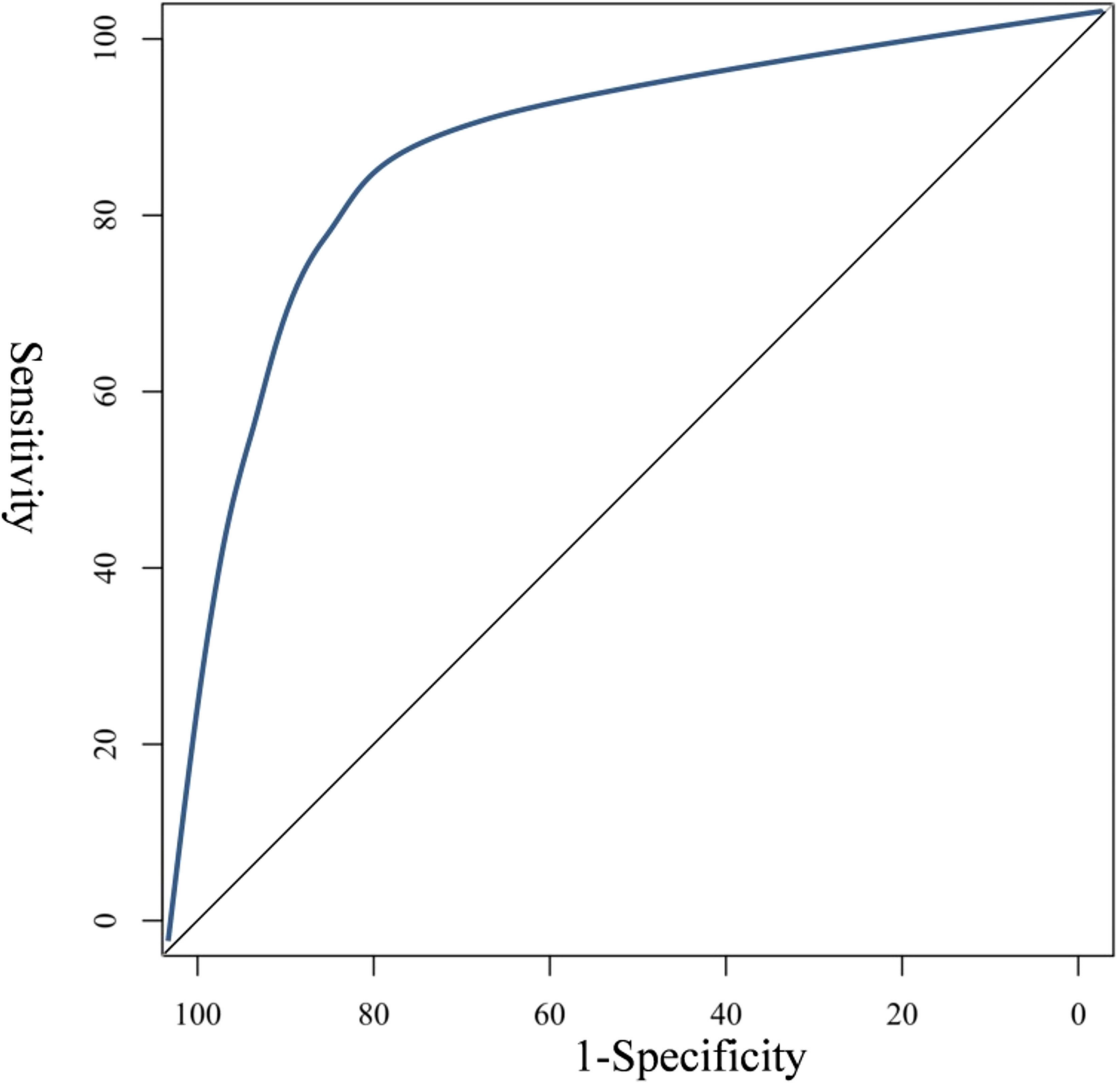
ROC curve of Nomogram prediction model.

Internal verification using the Bootstrap method showed that the predicted curve had a high degree of fitting with the standard model curve, and the average absolute error was 0.029, as shown in **Figure 4**. The results of ROC curve showed that AUC was 0.887, and 95%CI was 0.782–0.905, with corresponding specificity and sensitivity of 90.50% and 80.00%, respectively. See **Figure 5**. This indicates that the prediction accuracy of this Nomogram model is good.

## 4 Discussion

In recent years, there have been 1.7 million deaths due to NSCLC, and its incidence rate shows a rising trend every year. In the recurrent NSCLC, brain metastasis becomes the prominent focus, and its clinical manifestations include induced headache, vomiting, worsened cough, persistent tinnitus and language dysfunction, which seriously damage the quality of life of patients (5). At present, with the development of drugs and the emergence of advanced treatments, significant progress has been made in the field of treatment of NSCLC newly diagnosed as brain metastasis, and the treatment methods have been increased. However, the prognosis of NSCLC newly diagnosed as brain metastasis is still grim. Using more accurate detection markers to achieve early recognition and immediate intervention of influencing factors of newly diagnosed brain metastasis of NSCLC with poor prognosis is of great significance for significantly reducing the risk of death of patients, optimizing the disease outcome and improving the prognosis quality.

In this study, we found that NLR, mediastinal lymph node metastasis, CEA, extracranial metastasis, and GPS were the factors that affected the poor prognosis of NSCLC patients with newly diagnosed brain metastasis. Ren et al (10) found that an increase in NLR levels indicates a weakened anti-tumor immune response in NSCLC patients, and the proliferation rate of tumor cells will accelerate, thereby accelerating the progression of the disease. Sun et al (11) found that the metastasis of mediastinal lymph nodes indicates a higher risk of metastasis, and the involvement of regional lymph nodes has a significant impact on the subsequent progression of NSCLC newly diagnosed as brain metastasis. In our own pre - clinical studies, we have also explored the role of NLR in more depth. We conducted experiments on a small - scale cell line model and found that elevated NLR levels were associated with increased invasiveness of NSCLC cells. Specifically, when the NLR level was artificially increased in the cell culture environment, we observed enhanced migration and invasion abilities of NSCLC cells. This was accompanied by changes in the expression of certain key genes involved in the metastatic process, such as upregulation of matrix metalloproteinases (MMPs). These findings further support the role of NLR in the progression of NSCLC and its potential as a valuable biomarker for prognosis prediction. However, these results are preliminary and need to be further validated in larger - scale clinical trials. NLR is the platelet-to-lymphocyte ratio and an indicator for evaluating the inflammatory state. Initially, this indicator is used to assess the systemic inflammatory response in critically ill patients and patients with multiple traumas. Subsequently, in various systemic diseases, especially in the diagnosis of malignant tumors, quantitative evaluation of therapeutic effects, and prognosis prediction (10). As a key inflammatory cell, neutrophils not only have the ability to secrete vascular endothelial growth factor, which can significantly promote tumor angiogenesis, but also weaken the anti-tumor immune response of the body by inhibiting the activity of activated T lymphocytes (12). The increased NLR indicates that the anti-tumor immune efficacy of patients is weakened, and the proliferation of tumor cells will be accelerated, thus accelerating the progression of disease deterioration. The cutoff value for NLR (>2.94) was selected based on ROC curve analysis to optimize sensitivity and specificity in predicting poor prognosis. The most important route of metastasis of NSCLC is the lymphatic system, and the status of lymph node metastasis is crucial in the staging, treatment plan development and prognosis evaluation of NSCLC. The involvement of regional lymph nodes has a significant impact on the follow-up process of newly diagnosed brain metastasis in NSCLC (13). Sun J et al. (14) found that the metastasis of mediastinal lymph nodes indicates a higher risk of metastasis, indicating that there is a clear positive correlation between the size of lymph nodes and the frequency of metastasis. The study has found that (15)CEA detection has certain clinical application value in tumor diagnosis, differentiation of tumors to predict recurrence, etc. Studies have shown that CEA, a member of the immunoglobulin superfamily, belongs to the class of cell adhesion molecules, and its unique homophilic and xenophilic adhesion properties indicate that it plays a key role in the tumor metastasis mechanism (11). Positive cells in blood circulation, with their homogeneous adhesion, have the potential to promote the aggregation of tumor cells in the microcirculation system, and thus contribute to the formation of tumor plugs, which prolongs the residence time of tumor cells in the blood vessels and aggravates the risk of tumor metastasis (16). Low CEA levels in tumor tissues do not absolutely exclude the possibility of metastasis, whereas tumor tissues showing higher CEA levels are at significantly increased risk of metastasis. CEA levels are considered to be a key biomarker for the assessment of metastatic status of newly diagnosed brain metastatic tumors in NSCLC.

Studies (17) have found that extracranial metastasis has a significant correlation with the overall survival status of patients. Through the analysis of survival relationship, we found an obvious trend: With the increase in the number of involved organs and the accumulation of the number of metastatic lesions, the survival rate of patients shows a gradual decline. This finding confirmed that extracranial metastasis was an important factor affecting the survival and prognosis of patients with newly diagnosed brain metastasis from NSCLC (18). GPS, as a core indicator of prognosis in patients with malignant tumors, can accurately predict the overall survival rate and tumor-specific survival rate of patients before the start of treatment. Consistent with the findings by Agaoglu et al. (19), changes in GPS scores have become important indicators for predicting the prognosis of patients with NSCLC. The purpose of the study is to explore the prognostic factors of patients with NSCLC newly diagnosed with brain metastasis. Even in the background of mild inflammatory reaction, the potential cachexia tendency in patients may significantly affect the clinical outcome, indicating that the prognosis may be more unfavorable. Therefore, GPS is introduced as a key independent prognostic evaluation tool, which can effectively predict the survival rate of patients and has the ability to screen and identify potential cases of cachexia (20). For patients with high preoperative GPS scores, a more aggressive lymph node dissection strategy is recommended, i.e., a modest expansion of the range of lymph nodes to be dissected, in order to minimize the risk of potential tumor metastasis. In addition, the potential impact of genetic mutation status, maximum diameter of brain metastases, use of anti-vascular therapy drugs, and skull symptoms such as headache, dizziness, scalp itching, nausea, vomiting, and tinnitus on prognosis. Although these factors can provide valuable insights, their inclusion in this study is limited by the retrospective nature of our data collection. Future prospective studies should aim to incorporate these variables to further improve prognostic models and enhance their clinical applicability.

In addition, the application of column chart models in oncology and brain metastasis has also been widely studied. For example, Li’s (21) research directly discussed the application of column chart models in predicting the prognosis of NSCLC patients and validated their clinical value. Shapaer’s (22)study further explored the supportive role of column chart models in individualized treatment decisions for patients with brain metastases. These studies provide strong support for this study, demonstrating the effectiveness and reliability of the column chart model in predicting the prognosis of NSCLC patients newly diagnosed with brain metastases. In this study, we constructed a risk prediction nomogram model based on Logistic multiple regression analysis, which has excellent differentiation ability and accuracy, and provides a new perspective for medical staff to assess the prognosis of NSCLC after the new diagnosis of brain metastasis. By analyzing the contribution scores of each factor in the model, medical staff can explore how each factor independently and together acts on the prognosis and development of patients, and can formulate personalized prevention and care strategies to achieve more accurate interventions. Specifically, the performance of this risk prediction nomogram model in predicting the poor prognosis of patients with newly diagnosed brain metastasis of NSCLC has an AUC value of 0.887, a 95%CI of 0.782–0.905, and corresponding specificity and sensitivity of 90.50% and 80.00%, respectively. A study has found that (23), This result highlights the accuracy and reliability of the model in predicting the risk of postoperative infection, and provides a strong support for clinical decision-making, which has extremely high practical value. The incidence of SBM in NSCLC patients was 12.58%. The nomogram model developed in this study demonstrates high predictive accuracy (AUC = 0.887) and has the potential for broad clinical applicability. While the model was validated internally, future studies should assess its performance across different hospitals and regions to ensure generalizability. Additionally, the model can be integrated with other commonly used clinical variables, such as molecular biomarkers (e.g., EGFR mutation status) or imaging features, to further enhance its predictive power. This would allow for more personalized treatment planning and improved patient outcomes. While NLR is a well-established inflammatory marker, other related indicators such as dNLR, LMR, PLR, NPHR, PNI, SIRI, MLR, NMLR, and SII could provide additional insights into the inflammatory and immune status of patients. Future studies should consider incorporating these indicators to further refine the prognostic model and enhance its predictive accuracy. While several prognostic models for NSCLC with brain metastasis have been proposed, many of them focus on a limited set of clinical or molecular variables. For example, previous models often rely heavily on tumor size, number of brain metastases, and treatment modalities, but fail to incorporate inflammatory markers such as NLR or GPS, which have been shown to significantly impact prognosis. Our model integrates both clinical and inflammatory markers, providing a more comprehensive assessment of patient outcomes. Additionally, unlike some existing models that are limited by small sample sizes or lack of validation, our model was rigorously validated using the Bootstrap method, demonstrating high predictive accuracy (AUC = 0.887). This represents a significant improvement over previous models, which often lack robust validation or fail to account for the multifactorial nature of brain metastasis prognosis.

## 5 Conclusion

To sum up, NLR, mediastinal lymph node metastasis, CEA, extracranial metastasis and GPS are the risk factors for the poor prognosis of newly diagnosed brain metastasis in NSCLC. The risk factor model constructed based on these risk factors has excellent prediction value for the poor prognosis of newly diagnosed brain metastasis in NSCLC. Aimed at the above risk factors, targeted preventive measures were taken clinically to reduce the risk of newly diagnosed brain metastasis in NSCLC and improve the prognosis.

While this study provides valuable insights into the prognostic factors for NSCLC patients with newly diagnosed brain metastasis, it is important to acknowledge several limitations inherent in its retrospective design. First, the study relies on data collected from a single institution, which may limit the generalizability of the findings to other populations or healthcare settings. Second, the retrospective nature of the study introduces potential biases in sample selection and data collection. For example, patients with incomplete clinical data or those lost to follow-up were excluded, which may have introduced selection bias. Additionally, the reliance on electronic medical records for data collection may have resulted in missing or incomplete information, particularly for variables such as gene mutation status or detailed treatment histories. To address these limitations, future studies should consider a prospective, multicenter design to enhance the generalizability and robustness of the findings. Incorporating more comprehensive data collection methods, such as standardized protocols for recording clinical and molecular variables, would also help mitigate potential biases. Furthermore, future research could explore the integration of additional prognostic markers, such as molecular biomarkers (e.g., EGFR, ALK mutations) or imaging features, to further refine the predictive model. Finally, external validation of the nomogram model in independent cohorts would be essential to confirm its clinical applicability across different settings.

## Data Availability

The raw data supporting the conclusions of this article will be made available by the authors, without undue reservation.
